# Supplementary material for: TERT Expression Induces Resistance to BRAF and MEK Inhibitors in BRAF-Mutated Melanoma In Vitro
Source: Cancers (Basel). 2023 May 24;15(11):2888. doi: 10.3390/cancers15112888 (PMC10251862; doi:10.3390/cancers15112888)
Supplement: Supplementary file 1 [file cancers-15-02888-s001.zip › cancers-2348818-file S1.pdf]

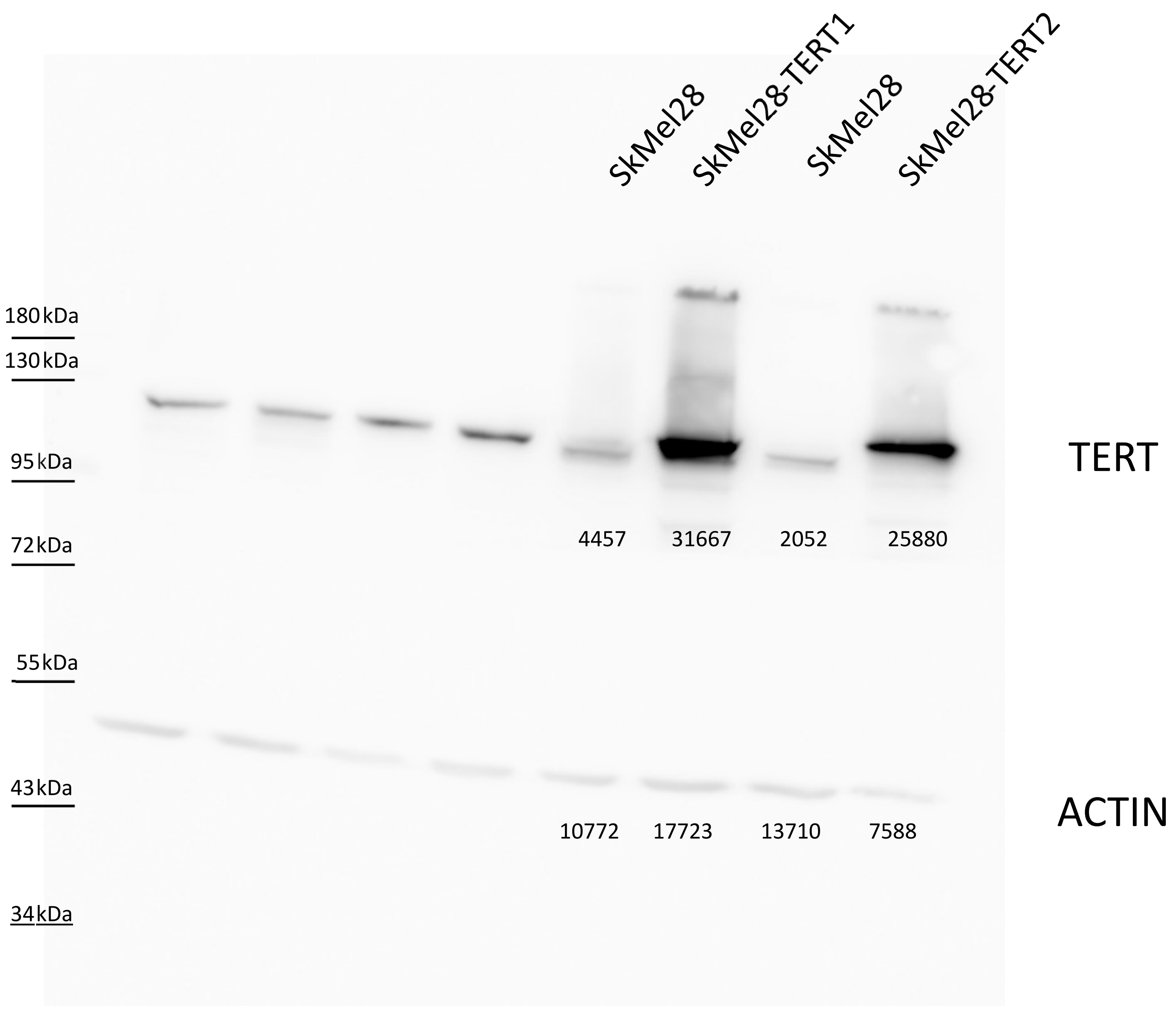

Figure 3A. Uncropped Western blot bands for Figure 3A. Quantification of western blots by ImageJ is indicated below the blot.

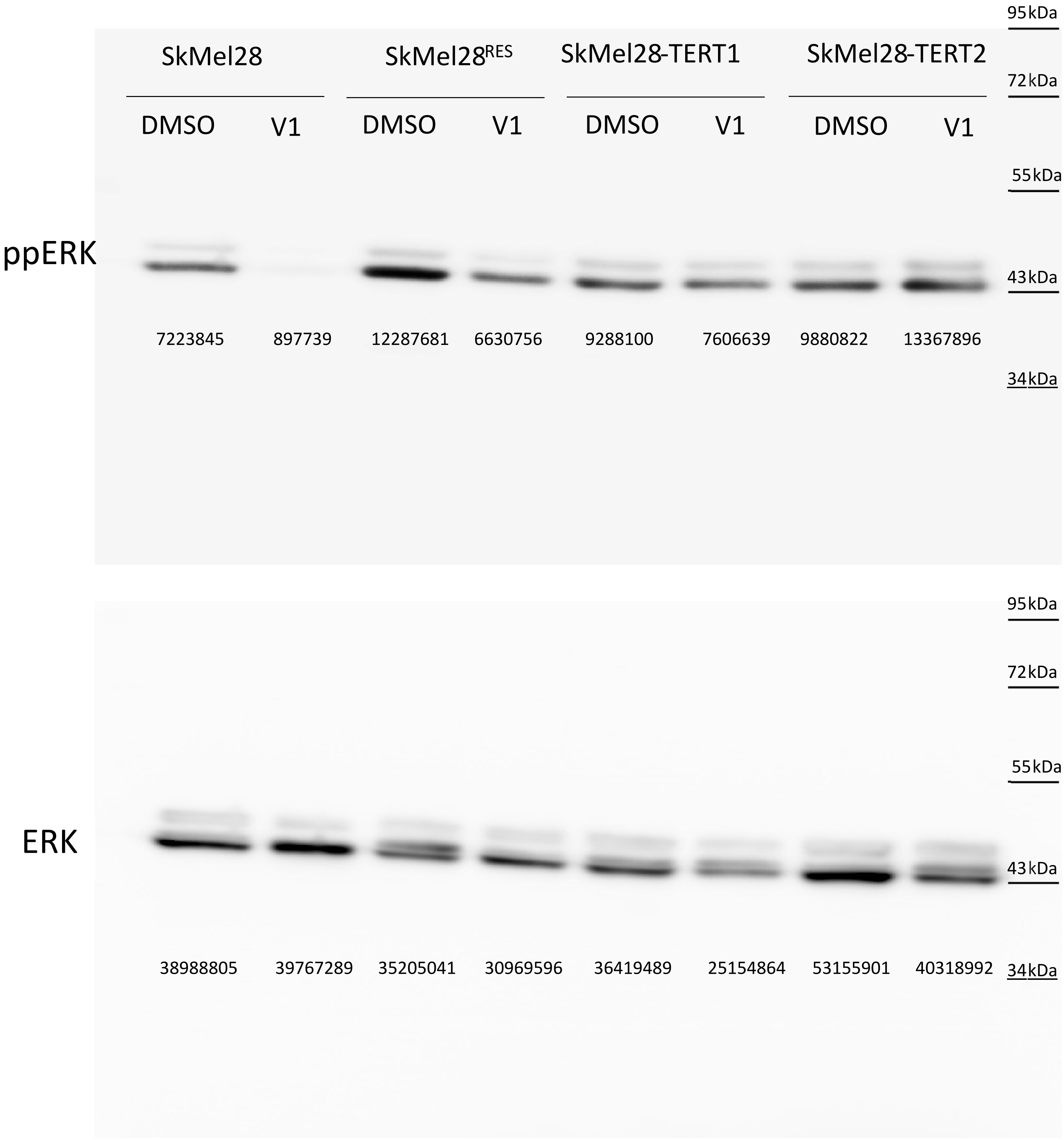

Figure 3E. Uncropped Western blot bands for Figure 3E. Quantification of western blots by ImageJ is indicated below each blot.

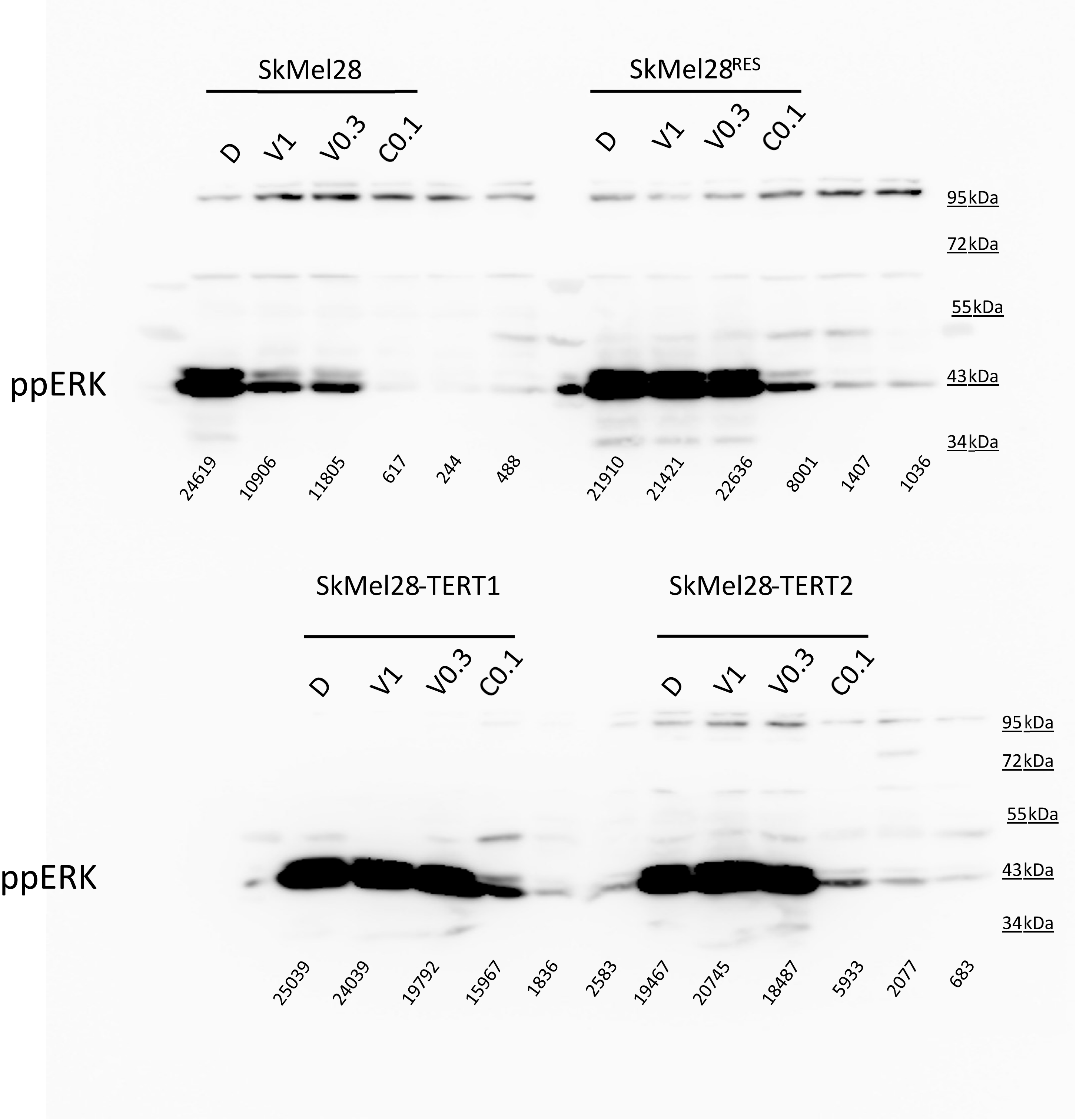

Supplementary Figure 1: Uncropped Western blot bands for Figure S1. Quantification of western blots by ImageJ is indicated below each blot.

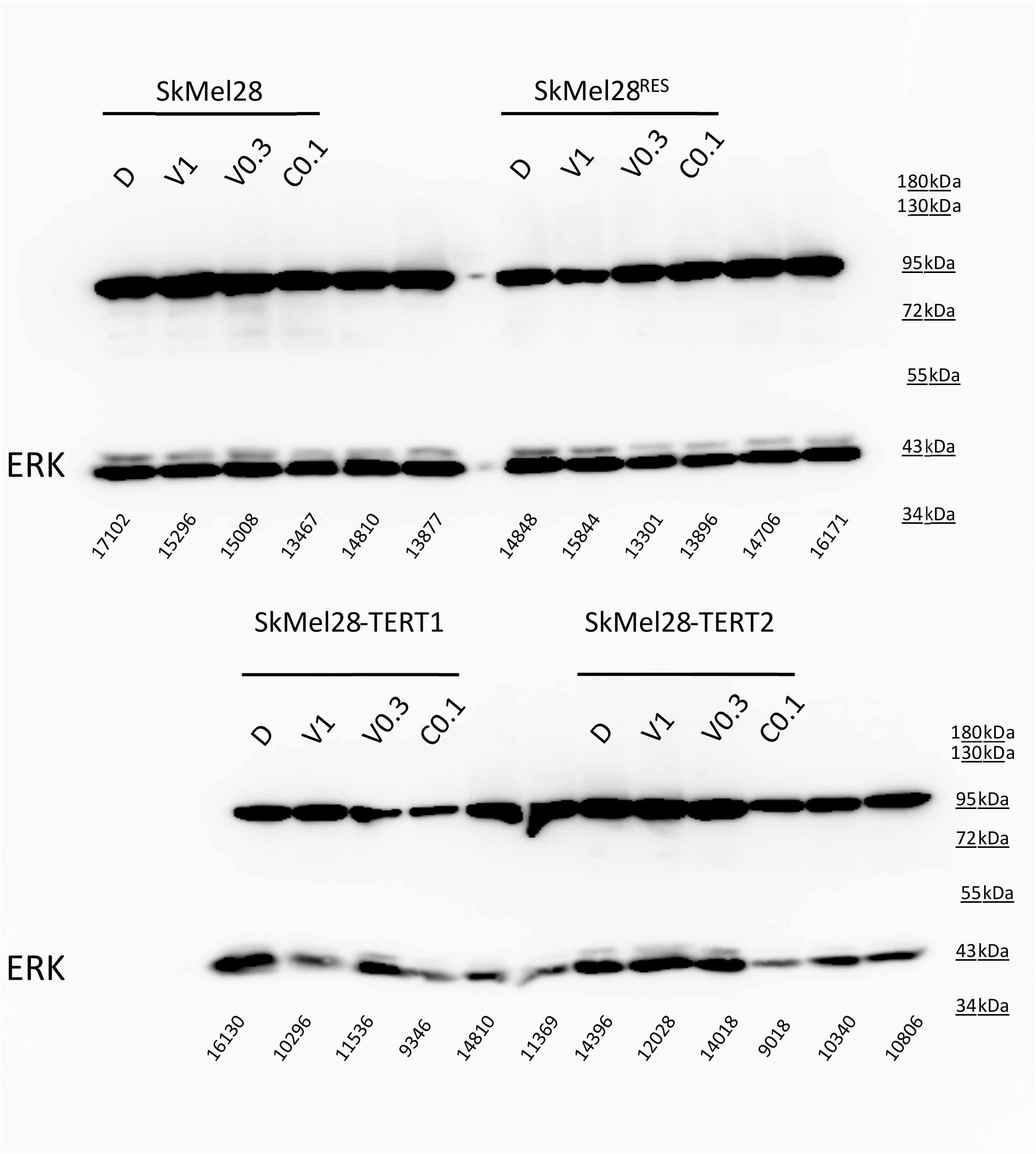

Supplementary Figure 1: Uncropped Western blot bands for Figure S1. Quantification of western blots by ImageJ is indicated below each blot.

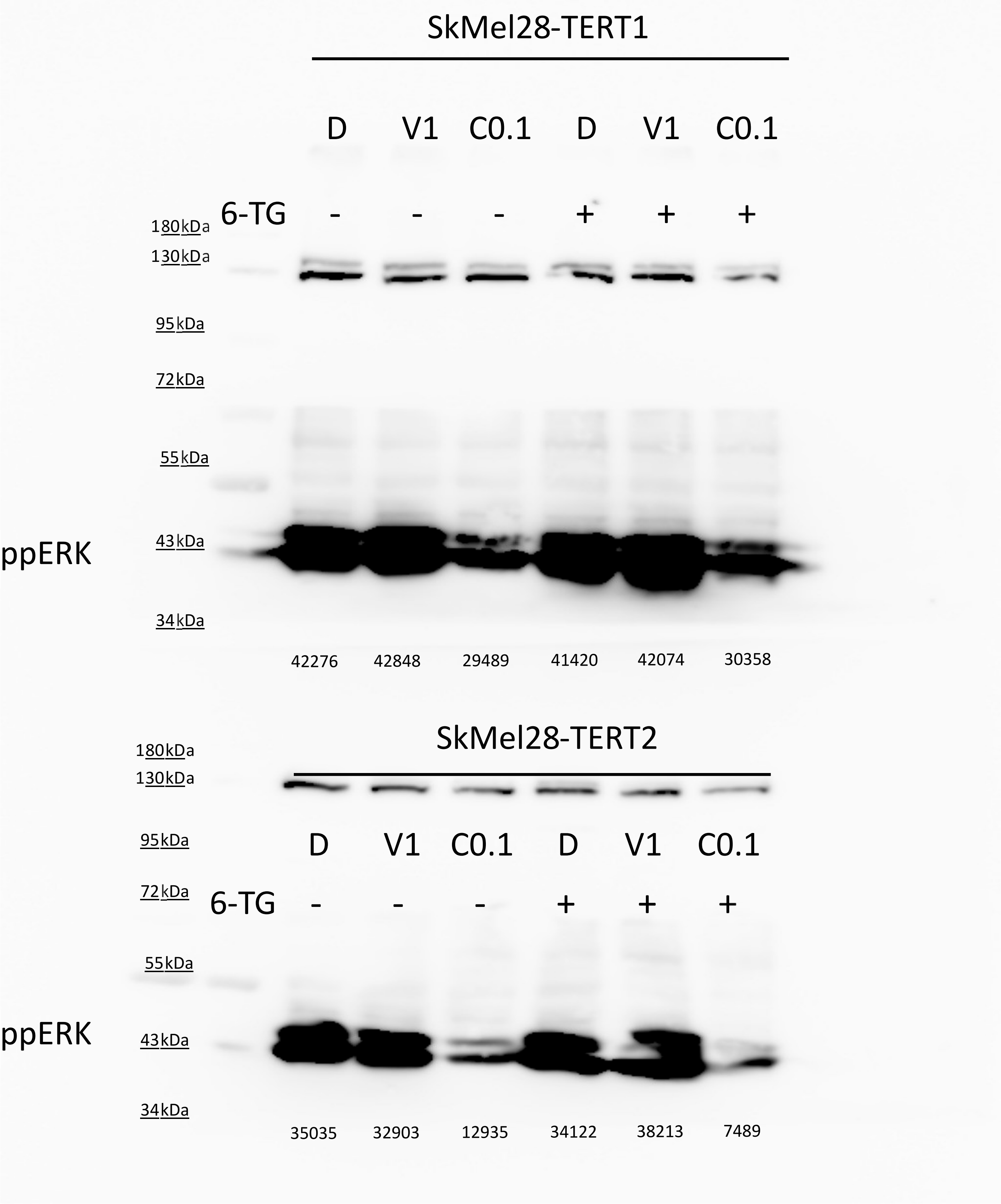

Supplementary Figure 2: Uncropped Western blot bands for Figure S2. Quantification of western blots by ImageJ is indicated below each blot.

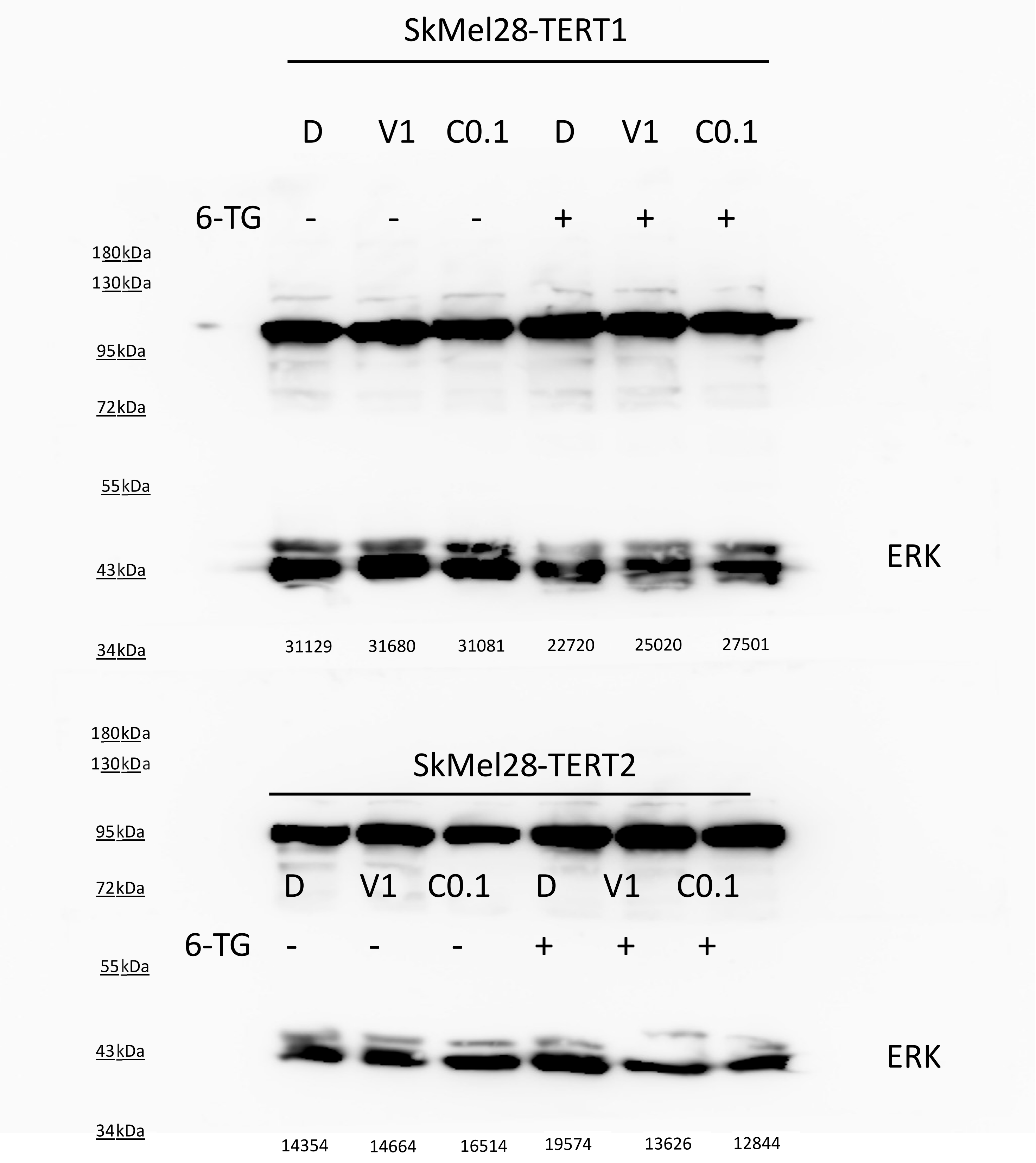

Supplementary Figure 2: Uncropped Western blot bands for Figure S2. Quantification of western blots by ImageJ is indicated below each blot.
