# Supplementary material for: TERT Expression Induces Resistance to BRAF and MEK Inhibitors in BRAF-Mutated Melanoma In Vitro
Source: Cancers (Basel). 2023 May 24;15(11):2888. doi: 10.3390/cancers15112888 (PMC10251862; doi:10.3390/cancers15112888)
Supplement: Supplementary file 1 [file cancers-15-02888-s001.zip › cancers-2348818-supplementary.pdf]

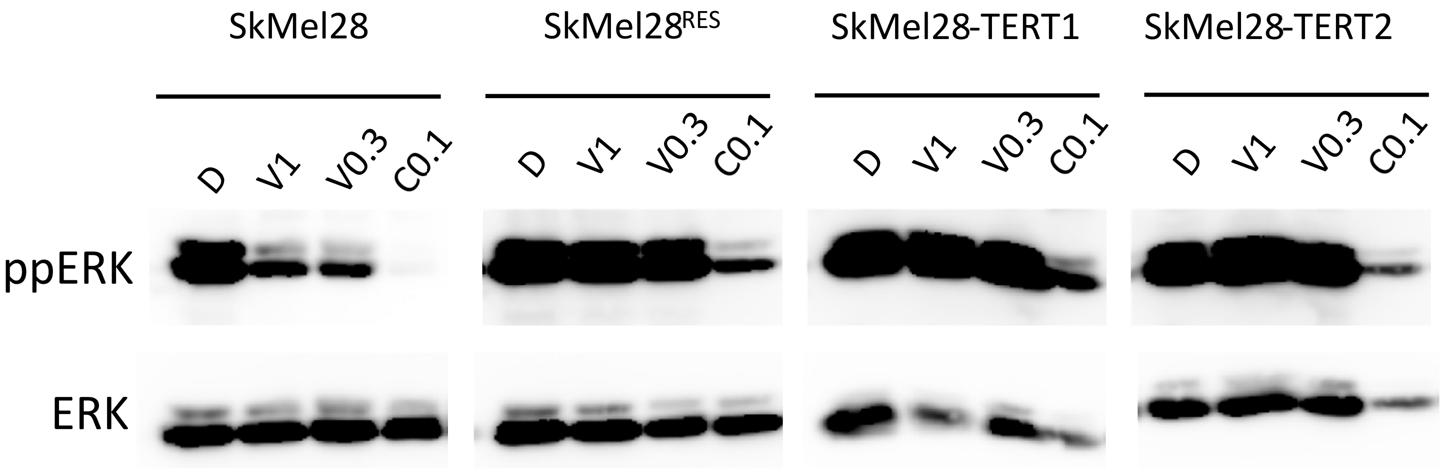

Supplementary Figure S1: TERT over-expression is associated with decreased sensitivity to BRAF and MEK inhibition The indicated cells were treated for 24 h with DMSO, vemurafenib 1 µM (V1) or 0.3 µM (V0.3) or cobimetinib 0.1 µM (C0.1) and the expressions of phosphorylated ERK and total ERK were assessed by Western blotting.

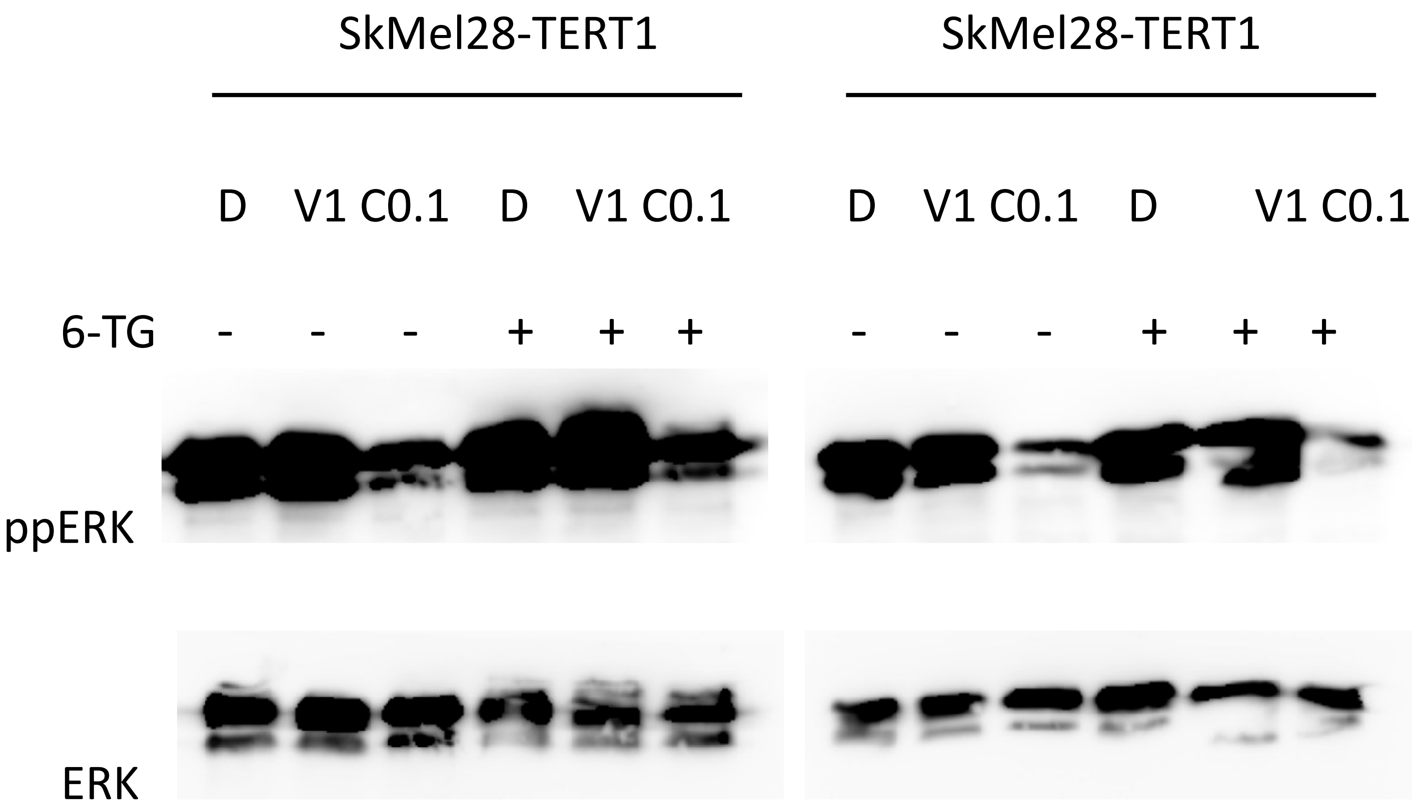

Supplementary Figure S2: The treatment with 6-thio-dG does not reduce ERK activation in cells treated with BRAFi or MEKi. The indicated cells were treated for 24 h with DMSO, vemurafenib 1 µM (V1), cobimetinib 0.1 µM (C0.1), 6-thio-dG 10 µM (6-TG) or the combination of inhibitors and the expressions of phosphorylated ERK and total ERK were assessed by Western blotting.

Supplementary Table S1: Tumor samples' characteristics

Patient id	BRAF/NRAS mutation status*	TERT promoter mutation	Relative mRNA TERT expression	BRAF and MEK inhibitors	PFS (months)
1442	BRAF V600R	-124 C>T	NA	Dabrafenib, trametinib	7,2
1865	BRAF V600E	-146C>T	NA	Pembrolizumab, dabrafenib, trametinib	2,6
1797	BRAF V600E	-124C>T	3,93	Vemurafenib, cobimetinib	4
1008	BRAF V600E NRAS Q61R	-124 C>T	111,71	Vemurafenib, cobimetinib	13,8
1217	BRAF V600E	-124 C>T	231,89	Dabrafenib, trametinib	5
768	BRAF V600E	-124 C>T	62,42	Dabrafenib, trametinib	8,5
1171	BRAF V600K	-124 C>T	NA	Vemurafenib, cobimetinib	37
1199	BRAF V600E	WT	NA	Dabrafenib, trametinib	5,6
563	BRAF V600E	-124 C>T	NA	Dabrafenib, trametinib	35,3
618	BRAF V600E	-124 C>T	29,92	Dabrafenib, trametinib	73,9
1807	BRAF V600E	-124C>T	NA	Dabrafenib, trametinib	7
1313	BRAF V600E	-146 C>T	NA	Vemurafenib, cobimetinib	4
1858	BRAF V600E	-124C>T	NA	Dabrafenib, trametinib	4
1102	BRAF V600E	-124 C>T	NA	Vemurafenib, cobimetinib	3,2
1555	BRAF V600E	-146 C>T	NA	Dabrafenib, trametinib	28
1196	BRAF V600E	WT	36,47	Dabrafenib, trametinib	11,1
1627	BRAF V600E	-146 C>T	NA	Dabrafenib, trametinib	11,3
857	BRAF V600K	-139-138 CC>TT	375,84	Dabrafenib, trametinib	3,9
1416	BRAF V600E	-139-138 CC>TT	NA	Dabrafenib, trametinib	9,1
1822	BRAF V600E	-124 C>T	NA	Dabrafenib, trametinib	2
1368	BRAF V600E	WT	NA	Dabrafenib, trametinib	23,6
1831	BRAF V600E	-124 C>T	NA	Dabrafenib, trametinib	30
1504	BRAF V600E	-124 C>T	1,23	Vemurafenib, cobimetinib	4,2
1016	BRAF V600E	-124 C>T	NA	Dabrafenib, trametinib	24
926	BRAF V600K	-146 C>T	293,82	Dabrafenib, trametinib	3,4
1746	BRAF V600K	-146 C>T	58,97	Dabrafenib, trametinib	13
1090	BRAF V600E	-124 C>T	3,79	Vemurafenib, cobimetinib	3,9
1833	BRAF V600E	WT	NA	Dabrafenib, trametinib	13,8
1903	BRAF V600E	-146 C>T	0,07	Dabrafenib, trametinib	3,1
1838	BRAF V600E	-146C>T	NA	Pembrolizumab, dabrafenib, trametinib	20,8
1447	BRAF V600E	-146 C>T	NA	Dabrafenib, trametinib	2,8
1316	BRAF V600E	-124 C>T	277,55	Dabrafenib, trametinib	1,1
1427	BRAF V600E	-146 C>T	NA	Dabrafenib, trametinib	3,2
1870	BRAF V600E	-139_138 CC>TT	NA	Dabrafenib, trametinib	3,1
1783	BRAF V600E	WT	NA	Dabrafenib, trametinib	13
1455	BRAF V600E	-125-124 CC>TT	NA	Vemurafenib, cobimetinib	4,8
1854	BRAF V600E	-124C>T	NA	Dabrafenib, trametinib	3,5
1579	BRAF V600E	-124 C>T	153,52	Vemurafenib, cobimetinib	1,9
1353	BRAF V600E NRAS Q61H	-124 C>T	NA	Vemurafenib, cobimetinib	2,8
1074	BRAF V600E	-124 C>T	NA	Encorafenib, binimetinib	23,7
707	BRAF V600K	-146 C>T	NA	Dabrafenib, trametinib	6,2
664	BRAF V600E	-125-124 CC>TT	405,04	Dabrafenib, trametinib	7,7
1363	BRAF V600E	-146 C>T	NA	Dabrafenib, trametinib	37,9
1413	BRAF V600E	-124 C>T	NA	Dabrafenib, trametinib	2,8
1863	BRAF V600E	-146C>T	NA	Pembrolizumab, dabrafenib, trametinib	5,3
1800	BRAF V600E	-124C>T	0		
1850	BRAF V600K	-146C>T	0		
1805	BRAF V600E	-146C>T	1453		

* NRAS is wild type unless otherwise specified; NA, not available (missing samples); PFS, progression free survival upon treatment with BRAF/MEK inhibitors; WT, wild type
